# Regulation of Skeletogenic Pathways by m6A RNA Modification: A Comprehensive Review

**DOI:** 10.1007/s00223-025-01367-9

**Published:** 2025-04-03

**Authors:** Ehsan Pashay Ahi

**Affiliations:** Organismal and Evolutionary Biology Research Programme, Faculty of Biological and Environmental Sciences, University of Helsinki, Viikinkaari 9, 00014 Helsinki, Finland

**Keywords:** Skeletal development, Skeletogenesis, m6A RNA modification, Epitranscriptomics, Signaling pathways, Post-transcriptional regulation

## Abstract

In the complex process of skeletal development, the significance of m6A RNA methylation—a predominant form of RNA modification—has not been fully explored. This review discuss how m6A RNA methylation plays an important, though not yet fully understood, role in regulating skeletal formation. It examines how m6A influences key signaling pathways essential for skeletal development and homeostasis, suggesting various possible interactions between m6A methylation and these critical pathways. While the exact mechanisms for many of these interactions remain to be elucidated, m6A RNA methylation is anticipated to be a key emerging regulator in skeletal structure development across vertebrates. Highlighting the need for further research, this overview provides an in-depth look at the potential regulatory interactions of m6A RNA methylation within skeletal system. Uniquely, this review is the most comprehensive compilation of evidence linking components of m6A RNA methylation to signaling pathways involved in skeletogenesis.

**Supplementary Information:**

The online version contains supplementary material available at 10.1007/s00223-025-01367-9.

## Introduction

N6-methyladenosine (m6A) is a prevalent RNA modification that extensively regulates RNA metabolism across the transcriptome. Found ubiquitously in eukaryotes, m6A methylation significantly impacts RNA processes like maturation, splicing, transport, degradation, and translation [1]. Initially documented in 1974 [2], the emergence of methylated RNA immunoprecipitation sequencing (MeRIP-Seq) renewed enthusiasm for m6A investigations [3]. The dynamic regulation of m6A modification involves the methyltransferase complex (MTC), including key enzymes such as METTL3, METTL14, METTL16, WTAP, RBM15, and ZC3H13, alongside other methyltransferases like METTL5-TRMT112 complex [4]. These writers catalyze m6A deposition on RNA, establishing modification patterns that influence RNA fate. Conversely, erasers like FTO and ALKBH5 mediate m6A demethylation (m6A removal), allowing reversible control over RNA stability and function. The levels of m6A modification are finely tuned by writers and erasers, while readers, such as RNA-binding proteins of the YTHDF and IGF2BP families, interpret these marks to regulate RNA decay, stabilization, splicing, transport, and translation [5]. Moreover, m6A modification influences various biological processes like self-renewal, differentiation, immune response, DNA damage response, tumorigenesis, environmental sensing and adaptation, and tissue development and morphogenesis [6–9].

The rise of m6A RNA modification has attracted considerable attention recently, owing to its involvement in a wide array of normal and pathological processes within skeletal tissues, encompassing tooth, bone, and cartilage [10, 11]. In the realm of skeletal cell differentiation, m6A modification has been implicated in steering the fate determination of mesenchymal stem cells (MSCs) towards osteogenic, adipogenic, or chondrogenic lineages [6]. Furthermore, m6A RNA modification assumes a critical role in the development and morphogenesis of skeletal tissues by regulating the expression of crucial genes and signaling pathways integral to skeletogenesis [9, 12]. Through meticulous post-transcriptional regulation of gene expression, m6A modification exerts influence over the proliferation, differentiation, and maturation of skeletal progenitor cells during both embryonic development and postnatal growth [13]. Additionally, m6A modification has been implicated in mechanotransduction processes within the skeletal system, where mechanical stimuli prompt cellular responses culminating in tissue remodeling and regeneration [14]. In this context, m6A modification serves as a molecular switch, translating mechanical cues into epigenetic and transcriptional alterations that modulate skeletal tissue homeostasis and repair. Moreover, dysregulation of m6A RNA modification has been linked to various skeletal cancers and diseases, including osteosarcoma, osteoporosis, and osteoarthritis [15]. Abnormal m6A modification patterns have been observed in skeletal tumor cells, impacting the expression of oncogenes, tumor suppressors, and genes involved in tumor progression and metastasis. Despite the rapidly growing research on the role of m6A RNA modification in skeletogenesis, mechanotransduction, and skeletal diseases, a comprehensive review elucidating the complex regulatory interplay between m6A modifications and signaling pathways involved in skeletal development and homeostasis is lacking. Hence, this review aims to provide a detailed molecular overview of all the relevant regulatory connections, illuminating the complex interplay between m6A RNA modification and skeletal tissue biology.

## Regulatory Connections Between m6A RNA Methylation and Major Skeletal Development Pathways

**Fig. 1:**
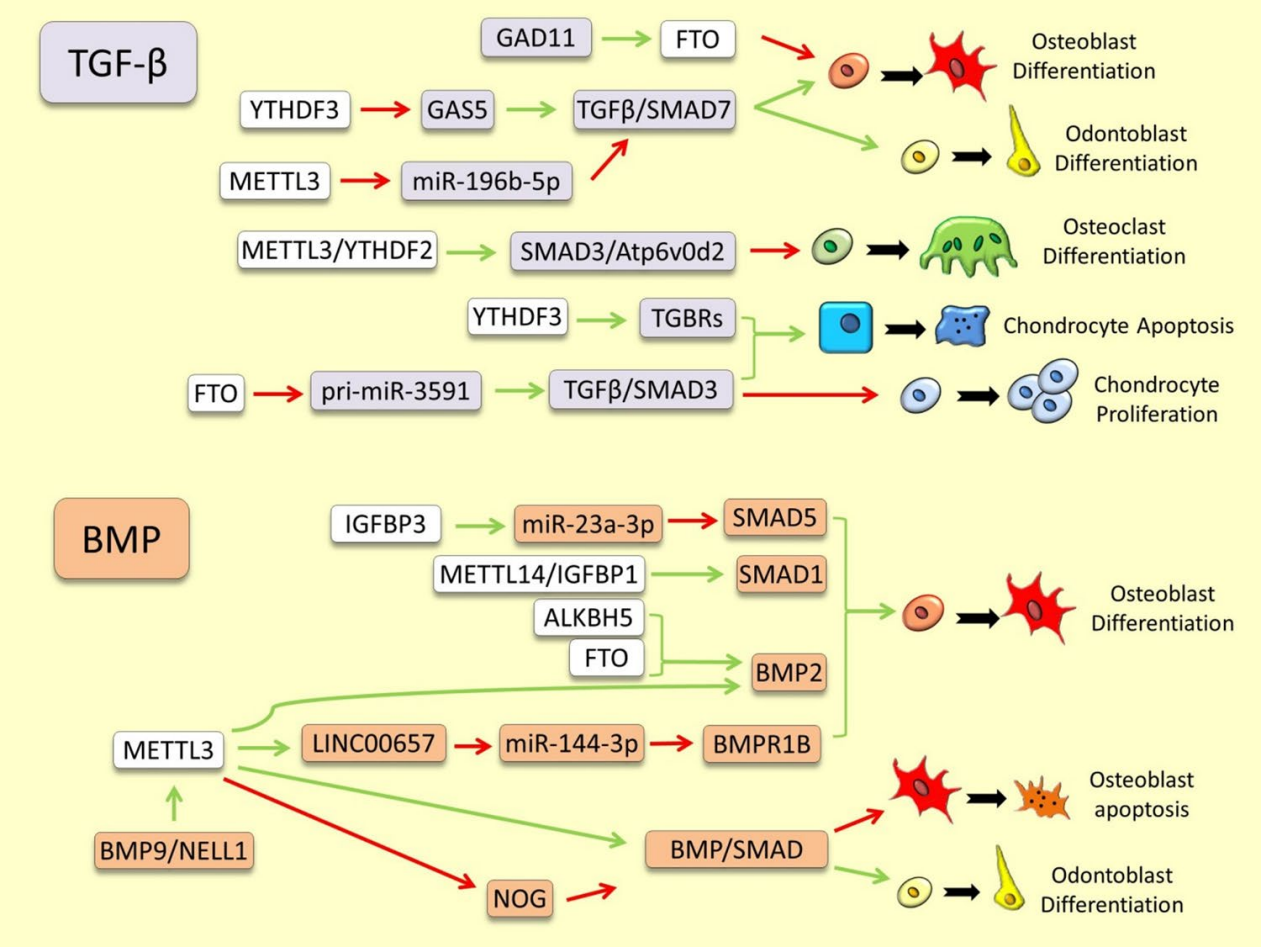
Examples of m6A RNA methylation dependent regulation of TGF-β and BMP signals in skeletal tissues. The green and red arrows indicate induction/enhancement and inhibition/degradation, respectively

**Fig. 2:**
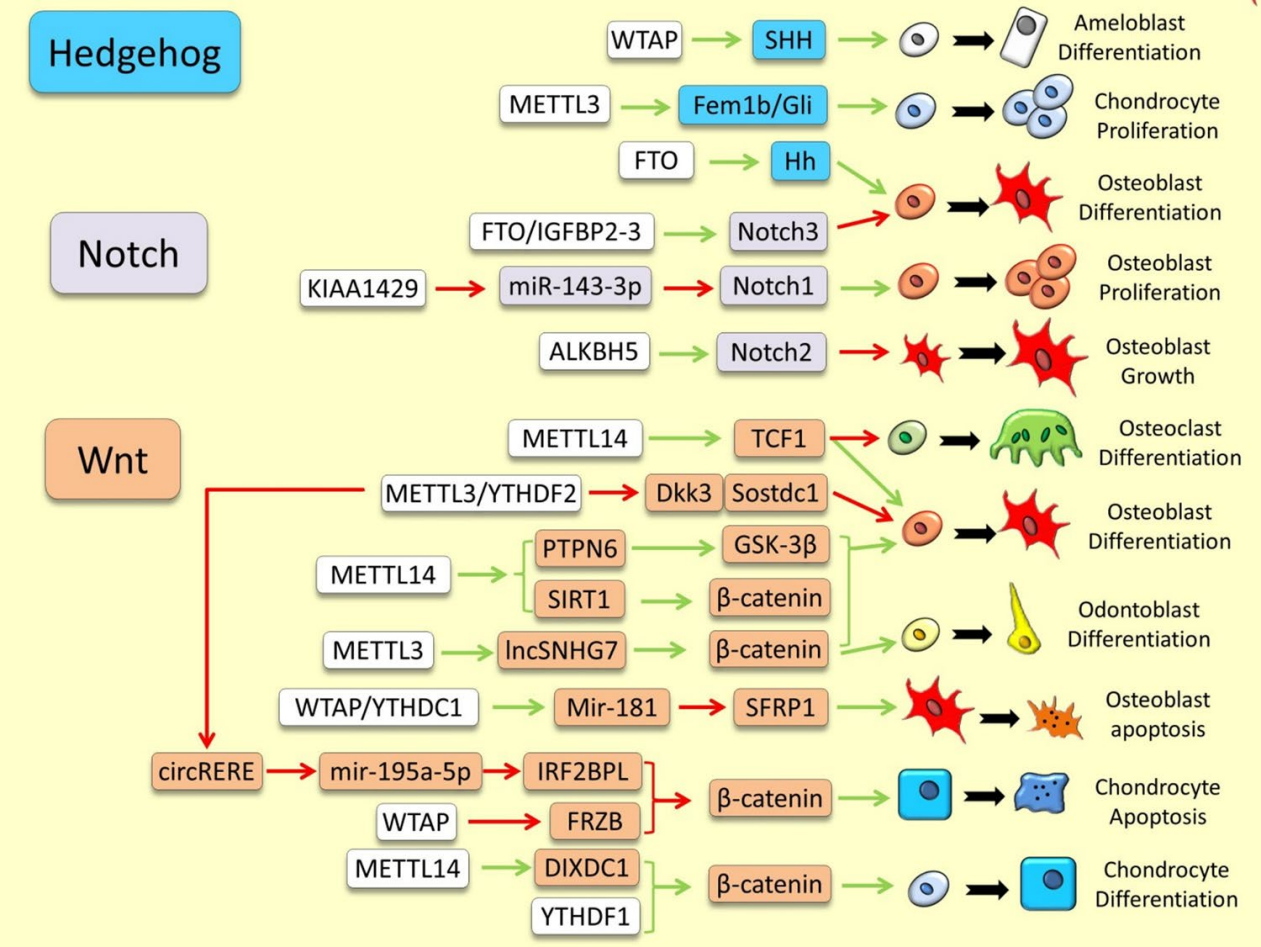
Examples of m6A RNA methylation dependent regulation of other developmental signaling pathways in skeletal tissues. The green and red arrows indicate induction/enhancement and inhibition/degradation, respectively

The most notable regulatory interactions between m6A RNA methylation modifiers and the pathway components presented in this section are depicted in Fig. 1 and 2, and are also outlined in Supplementary Table 1.

### Pathways Mediated by TGF-βs and BMPs Superfamily

The TGF-β superfamily comprises structurally related polypeptides conserved across the animal kingdom, synthesized as large precursors that undergo proteolytic cleavage, releasing mature active forms (e.g., BMPs) or mature latent forms (e.g., TGF-β) [16]. Secreted TGF-βs bind transmembrane receptors, whose binding and activity are regulated by various factors, including co-receptors (e.g., TGF-β type III receptor [TβRIII] and endoglin), integrins, and secreted modulators like decorin and latent TGF-β binding proteins (LTBP) proteins. These interactions transmit signals via intracellular SMAD proteins to regulate target genes and influence biological processes, including extracellular matrix (ECM) synthesis and skeletal remodeling [16–18]. Members of the TGF-β subfamily and their receptors are critical for skeletal structure development, morphogenesis and regeneration [19–23]. TGF-β1, a widely expressed member, plays a pivotal role in skeletogenesis, regulating skeletal metabolism and balancing bone formation and resorption [24, 25]. The effects of TGF-β pathways on skeletal morphogenesis involve ECM production modulation and regulation of major skeletogenic factors, including *BMPs, RUNX2, RANK, OPG*, and *TWIST1* [19, 24, 26].

Little is known about direct regulatory connections between components of m6A RNA modification and TGF-β signals in skeletal tissues, however, accumulating evidence indicates potential existence of such connection. For instance, *GDF11*, a member of the TGF-β superfamily and a major endogenous enhancer of odontoblast differentiation, has been found to be m6A hypomethylated at RNA level during ectopic osteogenesis [27]. Interestingly, an earlier study has shown that GDF11 can inhibit osteoblast differentiation and bone formation through direct up-regulation of *FTO* m6A demethylase [28]. A long non-coding RNA, *GAS5*, which is an upstream regulator of TGF-β signal with key role in various aspects of bone pathobiology, has been recently described as a downstream target of m6A RNA modification (negatively regulated by the m6A reader YTHDF3) [29]. Moreover, METTL3 mediated m6A methylation of *miR-196b-5p*, an upstream inhibitor of TGF-β signal, can enhance osteo/odontogenic differentiation [30]. This reveals an indirect synergistic regulatory connection between METTL3 and TGF-β signal during bone and tooth development. During bone remodeling, *Atp6v0d2*, a downstream target of TGF-β signal and a pre-osteoclast marker [31], has been found to be degraded by METTL3-dependent m6A methylation (guided by YTHDF2) which led to impairment of osteoclast function and bone remodeling [32]. Since both METTL3 dependent m6A methylation and TGF-β signal has inhibitory effects on *Atp6v0d2*, this may indicate another synergistic regulatory connection between them during bone remodeling as well. In inflammatory bone, increased METTL3 expression has been associated with hypomethylation of various components of TGF-β pathway, however, the regulatory connection between these processes has not been explored [33]. In inflammatory cartilage, an m6A reader, YTHDF3, has been shown to promote osteoarthritis via induction of TGF-β receptors, but again the detail underlying mechanism remained unknown [34]. Another study has found that FTO alleviates the inflammatory cartilage damage by m6A demethylation blocking of *pri-miR-3591* maturation which is essential for TGF-β/ Smad2/3 activation [35].

Bone morphogenetic proteins (BMPs), part of the TGF-β superfamily, signal via specific BMP receptors (BMPRs), activating SMAD proteins [36, 37]. Modulation occurs through extracellular and intracellular BMP antagonists, differential SMAD regulation, inhibitors, and feedback loops [36, 38]. In vertebrates, BMPs influence diverse skeletal structures; for example, Bmp4 signaling regulates tooth and neural crest-derived skeletal development [39, 40]. During early vertebrate development, ectodermal BMP signals interact with other morphogens to define gene expression domains [41, 42]. BMP signaling governs musculoskeletal cell differentiation and chondrogenesis. Its inhibition differently affects bone and cartilage formation across developmental stages, with elevated BMP levels favoring chondrogenesis over osteogenesis [43]. BMP signals regulate numerous transcription factors, including key skeletogenic factors, orchestrating dose-dependent gene expression networks [44].

Unlike TGF-βs, numerous studies had demonstrated direct and indirect regulatory connections between BMP signals and components of m6A RNA modification. Transcriptional activation of *BMP2* is essential for osteogenic differentiation of bone marrow mesenchymal stem cells (BMSCs); a crucial first step in bone formation and development. A recent study has found that inhibition of METTL3 function is required for m6A-mediated modification of *BMP2* in order to promote its expression during this process [45]. Although, another study has revealed an opposing role for METTL3 as well; i.e., METTL3 can enhance osteoblast differentiation through the methylation of *LINC00657* and in turn inhibition of *miR-144-3p* (a suppressor of *BMPR1B* signal during osteogenesis) [46]. Previously, it was also shown that increased expression of ALKBH5 can enhance ossification by promoting *BMP2* RNA demethylation [47]. A new finding has revealed that a toxicity induced bone loss utilizes METTL3-dependent molecular cascade to inhibit BMP pathway to inhibit osteogenesis (i.e., METTL3 suppression impaired the canonical BMP-SMAD signaling pathway) [48]. During tooth development, the METTL3-dependent m6A modification was found to be essential for inactivation of NOG, an inhibitor of BMP pathway, in order to promote odontoblast differentiation [49]. Interestingly, BMP pathway may also act at upstream of METTL3 during osteogenesis, as it is shown *NELL1*, a potent osteogenic factor target of BMP9, induces a METTL3-dependent molecular cascade leading to bone formation [50]. METTL14 mediated m6A modification of *SMAD1*, a key downstream mediator of BMP signaling transduction, is also required for increasing *SMAD1* RNA stability though a m6A reader (IGF2BP1) identification. Impairment of METTL14 can cause *SMAD1* RNA degradation resulting the inhibition of osteoblast differentiation [51]. In regenerating bone, IGFBP3-mediated m6A modification has been found as an upstream mechanism for regulating *SMAD5* during osteogenic differentiation [52]. In this, IGFBP3 guides m6A demethylation of *miR-23a-3p* and increases its stability, and in turn, *miR-23a-3p* suppresses *SMAD5* (downstream target of BMP2) and inhibits osteoblast differentiation [52]. Mechanical stress promotes osteogenic differentiation and the expression of *FTO* and this process is suggested to be mediated through FTO- dependent demethylation and increased expression of *BMP2* [53]. Taken together, these examples represent an emerging multi-faceted regulatory connection between components of m6A RNA modification and BMP signaling pathway during bone formation.

### Hedgehog Signaling Pathway

Hedgehog (Hh) signaling, among the earliest genetic pathways discovered for animal development, has been extensively studied in model species [54]. Vertebrates following whole genome duplication (WGD) and functional diversification encode three main Hh proteins: Desert hedgehog (Dhh), Sonic hedgehog (Shh), and Indian hedgehog (Ihh). Activated Hh proteins are released by the transmembrane protein Dispatched in generating cells and bind Ptch1 and Ptch2 receptors on target cells. Hh binding to Ptch frees Smoothened (Smo), another membrane protein, which interacts with Gli transcription factors to regulate target gene transcription [55]. The roles of Hh signaling components in skeletal structure development and as early initiators of cartilage differentiation are well-studied [56, 57]. Shh plays a pivotal role as an intermediary in skeletal structure formation, ensuring neural crest cell survival in the craniofacial skeleton [58]. Ihh, another Hh ligand with diverse skeletogenic roles, regulates palatogenesis, promotes chondrocyte proliferation and differentiation, and drives osteoblastogenesis and ossification [57, 59, 60].

A recent study in nervous system has revealed direct regulation of Shh signaling pathways by m6A RNA modification in which Mettl3 methylates *Ptch1* and *Gli2* RNAs and further regulates their RNA stability and translation [61]. In tooth development, Wtap is highly expressed in dental epithelium and its function is essential for amelogenesis and enamel formation. The Wtap function in enhancing ameloblast differentiation is mediated by Shh activity, i.e., through maintaining *Shh* mRNA stability [62]. During osteogenic differentiation, increased expression of FTO and reduced m6A methylation has been correlated with hypomethylation of Hh signaling components [63]. Similarly, in periodental tissues reduced expression of METTL14 was found to be correlated with differential expression of various Hh signaling components and osteogenic markers [64], however, in both studies, the direct regulatory connections between Hh components and these m6A modifiers have not been investigated [63, 64]. A recent study on chondrogenesis has demonstrated that Mettl3 can promote chondrocyte differentiation by increasing the stability of *Fem1b* and subsequently enhancing Gli activity [65]. Although research on the regulatory link between the Hedgehog (Hh) pathway and m6A RNA modification in skeletal development is currently limited, these findings hold promising potential for future studies.

### Notch Signaling Pathway

Notch proteins, exceptionally conserved transmembrane receptors, consist of three domains: extracellular, transmembrane, and intracellular [66]. Canonical ligands Jagged and delta-like (Dll) interact with Notch's extracellular domain, triggering the release of the intracellular domain. This liberated domain forms a complex with its transcriptional regulator CSL (RBPjk) to induce target gene expression, including Hes and Hey [66]. The canonical Notch signal, a remarkably simple molecular cascade, governs numerous biological processes, particularly cell fate determination. Beyond early somitogenesis, skeletal growth, and bone remodeling, it is involved in various aspects of skeletal development [67–69]. Notch signaling initially inhibits chondrocyte and osteoblast differentiation before initiating chondrogenesis [70]. By regulating genes like RANKL and OPG, it influences osteoclastogenesis during lineage commitment and maturation [67, 68, 71]. At different stages, Notch signaling hinders chondrocyte and osteoblast differentiation [68, 72].

During embryonic development *Mettl3*-dependent m6A methylation of *Notch1* and thereby inhibition of Notch pathway was found to be important for angiogenesis in skeletal tissues [9]. However, little is known about the direct m6A regulation of Notch pathway components in skeletal cells under normal condition. During osteogenic differentiation in mice, *Fto* expression is increased and in this process Fto-dependent demethylation leads to elevated expression of components within the Notch signaling pathway (e.g. *Notch3*) [73]. In addition, ALKBH5-induced m6A demethylation suppresses growth, migration, and invasion of osteosarcoma cells by enhancing the RNA stability of *NOTCH1* and *NOTCH2* [74]. Another study revealed that KIAA1429-dependent m6A methylation could repress *miR-143-3p* and increase the expression of its target gene, *NOTCH1*, leading to proliferation, migration and invasion of osteosarcoma cells [75]. These limited findings encourage further investigation of m6A mediated regulation of Notch signaling pathway in other skeletal cells under normal developmental processes.

### Wnt/β-Catenin Signaling Pathway

Wnts, a family of secreted glycoproteins, are crucial for processes like embryonic growth and morphological development, activating multiple signal transduction pathways. Their vital roles in skeletogenesis, potential for therapeutic skeletal regeneration, and master modulation through links with various morphogenic channels are well-recognized [76, 77]. Subtle variations in Wnt signal strength, periodicity, and duration influence developmental skeletogenesis, bone remodeling, and regeneration [76, 78]. Bone mineral density correlates with polymorphisms in Wnt pathway components [79]. In the canonical pathway, Wnts bind transmembrane Frizzled (FZD) receptors and co-receptors like LRPs (LRP5/6). This Wnt/LRPs/FZD complex initiates molecular events leading to β-catenin forming a nuclear regulatory complex with Lef and TCFs, modulating numerous target genes, including Runx2 and Ihh [80]. Wnt/β-catenin signaling also regulates transcription of bone homeostasis factors, RANKL and OPG [76, 81]. Its activity can be inhibited by transmembrane proteins (Kremen1/2, Ror2, Ryk) and secreted Wnt antagonists (Dkks, Sfrps, Wif1, Sost) [82, 83]. Canonical Wnt signaling governs cranial neural crest cell epithelial-to-mesenchymal transition, breakdown, and migration to cranial regions [84].

Although the regulatory links between m^6^A RNA modification and the Wnt/β-catenin signaling pathway are perhaps the most thoroughly investigated in the field of epitranscriptomics of skeletal biology, the details of their molecular interactions have only been disclosed recently. In a pioneering study of bone cancer, METTL3 has been identified to increase m6A levels of *LEF1*, a major downstream transcription factor of the Wnt pathway [85], and upregulates the Wnt/β-catenin signaling pathway [86]. A later study found that FTO-dependent demethylation could reduce *DAC1* mRNA stability, a negative regulator of the Wnt/β-catenin pathway, to accelerate the proliferation of osteosarcoma cells [87]. In bone under normal condition, it has been also shown that m6A modifiers can act at upstream of Wnt pathway; as METTL14-dependent m6A methylation of *TCF1* mRNA, another major transcription factor in the Wnt pathway, was found to be crucial for enhancing its stability during osteogenesis [88]. This new finding demonstrated that the m6A mediated enhancement of *TCF1* mRNA stability favors osteoblast differentiation whereas suppresses osteoclast differentiation [88]. In addition, METTL3-dependent m6A methylation has been recently suggested to be crucial for homeostasis and other biological functions of osteoblasts, such as ribosomal and mitochondrial function, through activation of the Wnt/β-catenin signal [89]. Interestingly, LEF1 was not the target of METTL3 during this activating process, and instead, METTL3-dependent m6A methylation of two Wnt antagonists (*DKK3* and *SOSTDC1*) and their degradation via YTHDF2 was the underlying molecular mechanism for activation of the Wnt/β-catenin signal in osteoblasts [89]. During endodontic regeneration, METTL3 can activate the Wnt/β-catenin signaling pathway by regulating the m6A modification and expression of *lncSNHG7* to enhance the osteogenic/odontogenic differentiation [90]. Another new finding revealed that WTAP-dependent m6A methylation of two miRNAs (*miR-181a* and *miR-181c*) is required for their maturation (after recognition by YTHDC1) and their function in promoting the osteogenic differentiation. Importantly, the *miR-181a/c* function is mediated by suppression of their target gene, *SFRP1*, which is an antagonist of the Wnt signaling pathway and an inducer osteoblasts and osteocytes apoptosis. This represents a novel regulatory connection between WTAP-dependent m6A methylation and the Wnt signaling pathway in promoting bone formation by reduction of apoptosis [91]. An indirect activation of the Wnt signaling pathway by METTL14-dependent m6A methylation was also reported during osteoblast differentiation [92, 93]. For instance, METTL14-dependent m6A methylation increases *PTPN6* mRNA stability, which is an interacting partner of another major WNT component (GSK-3β), and thus amplifies the Wnt signaling activity [92]. Similarly, Mettl14-dependent m6A methylation increases *Sirt1* mRNA stability, which in turn enhances osteoblast differentiation and reduces osteoclast differentiation [93]. While the Sirt1 mediated suppression of osteoclastogenesis is mediated through NF-κB pathway, the Sirt1 mediated induction of osteoblast differentiation is mediated through activation of the Wnt signaling pathway (Sirt1 deacetylates β-catenin and protects it from ubiquitination) [94]. These studies show that three main writers of m6A methylation, METTL3, METTL14 and WTAP play role in modulation of the Wnt signaling in bone.

In cartilage, YTHDF1 m6A reader enhances chondrogenic differentiation via direct activation of the Wnt/β-catenin signaling after facilitating β-catenin mRNA translation [95]. Under inflammatory condition, WTAP-dependent m6A methylation of *FRZB* mRNA, an inhibitor of the Wnt pathway, leads to its degradation and thus excessive activity of the Wnt pathway and chondrocyte apoptosis [96]. Previously, another regulatory axis was also described in cartilage under inflammatory condition in which METTL3-dependent m6A methylation of *circRERE* leads to its degradation (via YTHDF2) followed by an increase *miR-195a-5p* (downstream target of *circRERE*) and inhibition of *IRF2BPL* unbiquitination factor (downstream target of *miR-195a-5p*). The inhibition of IRF2BPL leads to nuclear elevation of β-catenin and excessive induction of the Wnt pathway resulting chondrocyte apoptosis [97]. In regenerating cartilage, a *Mettl14*-dependent m6A methylation has been found to increase *Dixdc1* mRNA stability, a positive regulator of Wnt pathway, which in turn impaired chondrocyte differentiation and regeneration [98]. Taken together, these examples also demonstrate the involvement of various m6A components during chondrogenesis through modulation of canonical Wnt/β-catenin pathway, however, unlike bone; most of these m6A-mediated activations of the Wnt pathway appeared to have apoptotic outcome in cartilage.

## Regulatory Connections Between m6A RNA Methylation and Growth Factors Mediated Signals

**Fig. 3:**
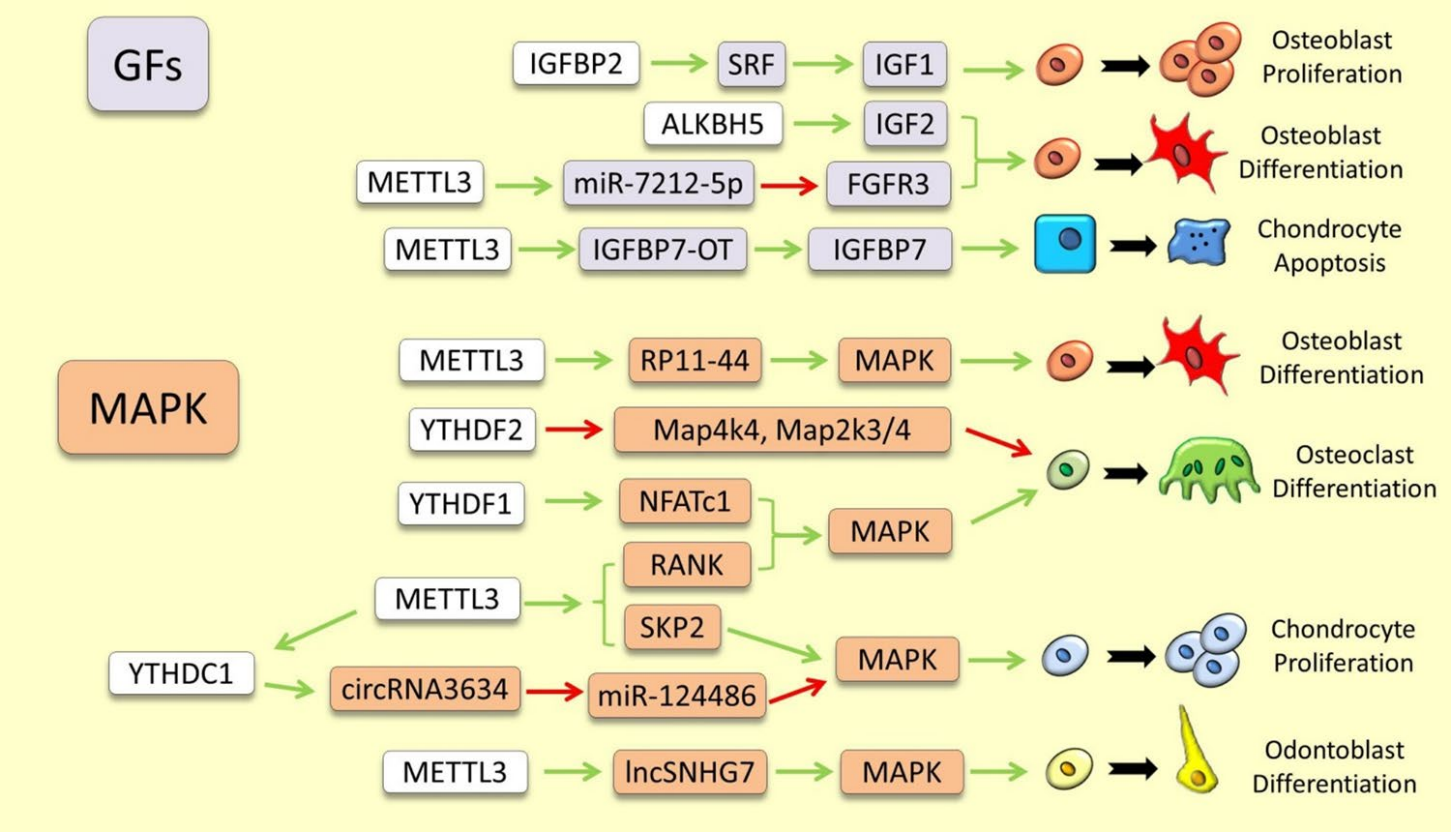
Examples of m6A RNA methylation dependent regulation of growth factors and MAPK signaling pathways in skeletal tissues. The green and red arrows indicate induction/enhancement and inhibition/degradation, respectively

**Fig. 4:**
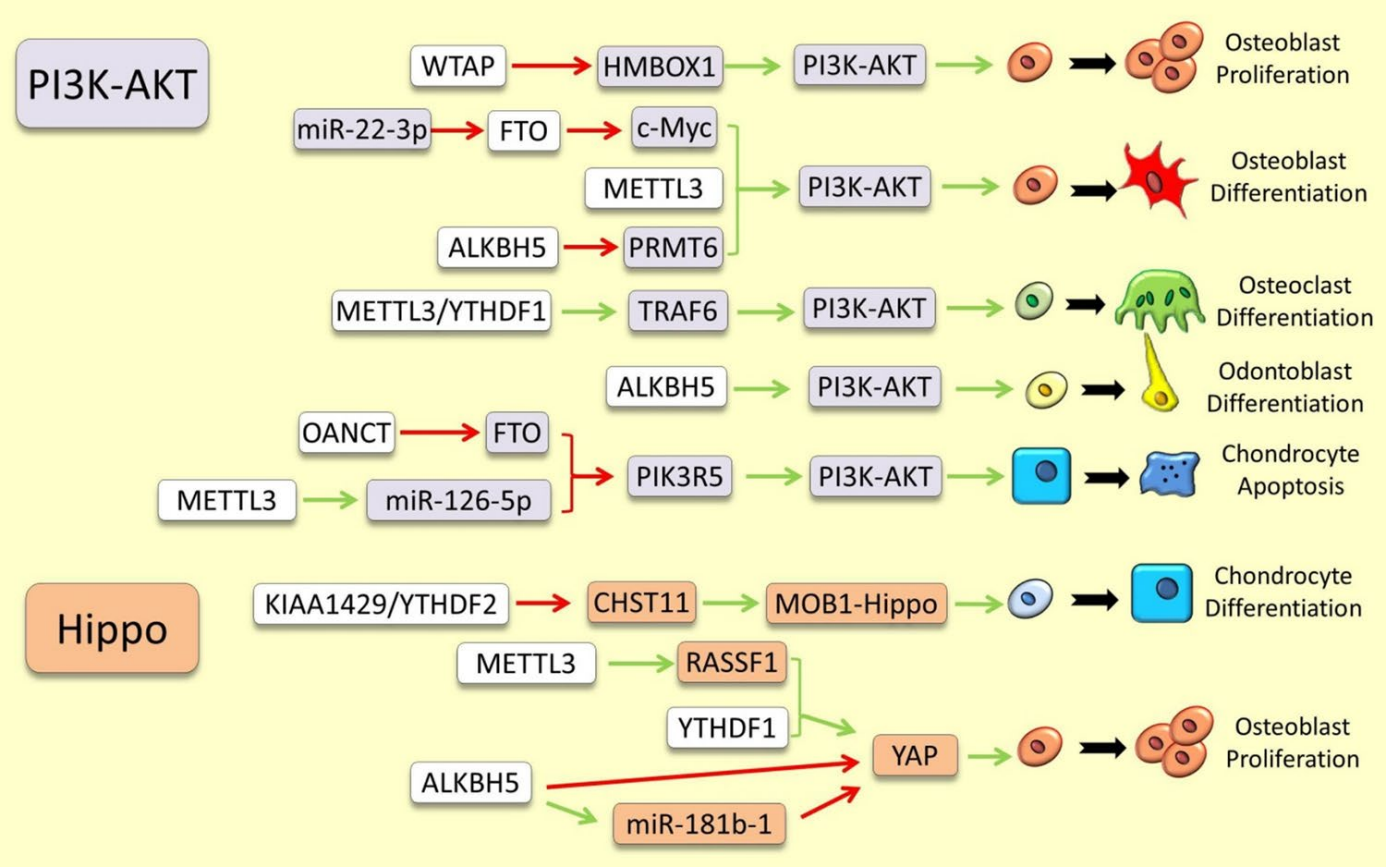
Examples of m6A RNA methylation dependent regulation of PI3K-AKT and Hippo signaling pathways in skeletal tissues. The green and red arrows indicate induction/enhancement and inhibition/degradation, respectively

The most notable regulatory interactions between m6A RNA methylation modifiers and the pathway components presented in this section are depicted in Fig. 3 and 4, and are also outlined in Supplementary Table 2.

### Growth Factor Signaling Pathways

Fibroblast growth factors (FGFs), a large family of primarily paracrine ligands, activate numerous conserved signaling pathways and are crucial in vertebrate development and various biological processes [99, 100]. FGFs signal via FGF receptors (FGFRs), a tyrosine kinase family with isoforms generated by tightly regulated alternative splicing, vastly exceeding the number of encoding genes [101]. FGFs and FGFR isoforms exhibit distinct spatiotemporal expression patterns during development, and their disruption is linked to developmental and morphological abnormalities [102–106]. FGF-regulated pathways influence endochondral and intramembranous bone development, chondrogenesis, and bone mechanical sensing [102, 107, 108]. Insulin-like growth factors (IGFs), initially identified in the musculoskeletal system, mediate growth and differentiation via conserved cascades involving IGFs, IGF receptors, IGF binding proteins (IGFBPs), and IGFBP proteases [109]. IGFs bind activated receptors, triggering gene regulatory signals through MAPK and PI3K-AKT pathways. IGFBPs modulate IGF bioavailability, impacting IGF functions. Present in all tissues, IGFs are vital for homeostasis, development, and tissue survival [110–112]. Predominant in bones, IGFs regulate mineralization, differentiation, and formation while affecting chondrocyte proliferation, differentiation, and apoptosis [113, 114].

Overall, there is limited knowledge of the direct regulatory links between IGF and FGF signaling pathways and m6A RNA modification in skeletogenesis. It has been shown that the demethylation of *IGF-2* by ALKBH5 promotes its expression, which in turn promotes osteogenesis and ossification [115]. Also, the initial demonstration of a regulatory link between FGF signaling and m6A RNA modification was identified in a study by Mi et al. (2020). This research found that Mettl3-dependent m6A methylation facilitates the maturation of *miR-7212-5p*. Subsequently, *miR-7212-5p* inhibits osteoblast differentiation by targeting *Fgfr3*, providing a clear example of the interaction between these pathways [116]. Despite the scarcity of such direct regulatory connections, the IGF binding proteins (IGFBPs) are well recognized for their role as m6A reader during skeletal formation. For instance, in dental pulp stem cells (DPSCs), METTL3 enhances m6A-dependent mRNA stability of *ACLY* and *SLC25A1*, guided by IGF2BP2/3 readers and thereby induces their odontoblastic differentiation [117]. In bone, IGF2BP2 enhances mRNA stability of *SRF*, an osteogenic factor, and promotes osteoblast proliferation and osteogenesis [118]. Moreover, a regulatory axis of IGFBP3/m6A-dependent stabilization of *miR-23a-3p*, an upstream inhibitor of SMAD5 protein, was recently described as an suppressor of bone regeneration by blocking osteoblast proliferation and differentiation [52]. The METTL14 can also regulate osteogenesis of bone marrow mesenchymal stem cells via IGF2BP1/2/3 recognition of m6A-methylated *Beclin-1* mRNA. This recognition leads to increased translation of *Beclin-1* mRNA and consequently suppression of osteoclastogenesis while enhancing osteoblast differentiation [119]. In cartilage, METTL3-dependent m6A methylation improved the stability of *IGFBP7-OT* (a lncRNA controlling *IGFBP7* expression) and in turn *IGFBP7-OT* suppresses the occupancy of DNMT1/DNMT3a on the *IGFBP7* promoter and upregulates its expression. The increased *IGFBP7* expression leads to chondrocyte apoptosis and progression of osteoarthritis [120]. These instances encourage additional exploration into whether other elements of the IGF signaling pathway are influenced by m6A RNA modification during skeletogenesis, or if the roles of IGFBPs in these processes operate independently from their downstream IGF signaling pathway.

### Signals Mediated by MAPKs

The mitogen-activated protein kinases (MAPKs), a conserved family of serine/threonine kinases, are essential for transmitting external signals into cells via membrane receptors [121]. MAPKs include key regulatory pathways such as extracellular signal-regulated kinase (ERK), c-Jun NH2-terminal kinase (JNK), and p38 MAPK. While growth factors are the primary activators, each MAPK cascade transmits distinct cellular signals associated with apoptosis, stress responses, differentiation, and growth [122–124]. Members of the Ap-1 complex (c-Jun and c-Fos heterodimer), which regulate gene expression during osteoblast differentiation, are specific MAPK targets. Notably, MAPK cascade activation is crucial for the development of mesodermal derivatives, including the skeleton and dentition. In addition, bone mechanotransduction is mediated by JNK and ERK, which induce Ap-1 transcriptional activity [125].

The first study investigating the regulatory relationship between m6A RNA modification and MAPK signaling was performed in the context of inflammatory bone conditions. It demonstrated that depletion of METTL3 in response to inflammation amplifies the MAPK signaling pathway evidenced by increased phosphorylation levels of ERK, p38, and JNK [126]. Another study under normal condition also showed that METTL3 can activate the MAPK signaling pathway, after phosphorylation of ERK, JNK and p38, by regulating the m6A modification a lncRNA (*RP11-44*), thereby enhancing the osteogenic differentiation [50]. During osteoclastogenesis, the expression of METTL3 and m6A methylation are elevated; this results an increase in RNA stability of *NFATc1* and enhancement of MAPK signal promoting osteoclast differentiation and bone resorption [32]. These studies suggest that METTL3-dependent activation of MAPK signaling is involved in both bone formation and inflammatory response. The MAPK signaling can also be regulated by m6A readers during skeletogenesis. For instance, Ythdf2 has a negative regulatory role in osteoclastogenesis and the inflammatory response in bone via m6A mediated mRNA degradation of several MAPKs (*Map4k4*, *Map2k3*, and *Map2k4*) [127]. In cartilage, increased expression of YTHDF1 during inflammation has been to show opposite role of enhancing osteoclastogenesis and bone resorption by improving *RANK* mRNA stability and in turn activating MAPK signal at its downstream [128]. In addition, SKP2 is an upstream activator of MAPK signal during chondrocyte proliferation [129], and a recent study demonstrated that *SKP2* mRNA is among m6A methylated genes during cartilage inflammation (probably through METTL3 dependent m6A methylation) [130]. This may suggest a METTL3-dependent regulation of MAPK signaling through SKP2 in inflammatory cartilage opposite to its role during bone inflammation. Under normal condition in cartilage, METTL3/YTHDC1-mediated m6A modification of *circRNA3634* regulates chondrogenesis through *miR-124486–5-MAPK1* axis. In this process, METTL3 m6A methylation of *circRNA3634* leads to its nuclear export guided by YTHDC1 and then *circRNA3634* acts as a molecular inhibitor of *miR-124486–5*, leading to increased *MAPK1* expression and enhanced chondrocyte proliferation, differentiation and migration [131]. In tooth, METTL3 dependent m6A methylation regulates expression of *lncSNHG7* to enhance the odontogenic differentiation and components of MAPK pathway were found to be the downstream targets of *lncSNHG7* during this process [90]. These examples illustrate the extensive regulatory connections between m6A RNA modifiers and MAPK signals in various skeletal cells and skeletogenesis.

### PI3K-AKT Pathway

The PI3K-AKT signaling pathway is categorized as a growth factor-mediated signaling pathway due to its activation by various growth factors such as insulin, IGF-1, and epidermal growth factor (EGF). Upon stimulation by these growth factors, PI3K is activated, leading to the subsequent phosphorylation and activation of AKT, which then regulates downstream molecular processes (e.g. mTOR signal) involved in cell growth, proliferation, and survival [132]. The PI3K-AKT pathway plays a pivotal role in skeletogenesis and the maintenance of skeletal tissues, including bone, cartilage, and tooth [132–134]. This pathway is intricately involved in regulating various aspects of skeletal development, homeostasis, and remodeling. In osteoblasts, the PI3K-AKT pathway promotes cell proliferation, and differentiation [135]. Activation of AKT by PI3K signaling leads to increased expression of osteogenic genes and enhances the mineralization process, contributing to bone formation [135, 136]. In osteoclasts, the PI3K-AKT pathway regulates cell survival, activity and differentiation, and its inhibition leads to impaired bone resorption, highlighting its crucial role in bone remodeling [137]. Furthermore, in chondrocytes, the PI3K-AKT pathway influences proliferation, differentiation, and matrix synthesis [136, 138]. Activation of AKT has been shown to promote chondrocyte proliferation and inhibit their hypertrophic differentiation, thereby regulating cartilage growth and development [138]. Overall, the PI3K-AKT pathway emerges as a key player in orchestrating the complex balance between osteoblast, osteoclast, and chondrocyte activities, thereby influencing skeletogenesis and skeletal tissue homeostasis through a variety of mechanisms.

The crosstalk between the PI3K-AKT pathway and components of m6A RNA modification is one the most studied regulatory connections within the field of epitranscriptomics of skeletal tissues. In bone, *miR-451a* represses the malignant progression towards osteosarcoma via blocking YTHDC1-mediated m6A methylation of *PDPK1* (an activator of AKT), and subsequently inhibiting AKT/mTOR pathway. This repressive effect on malignant progression is acting as a gatekeeper of excessive proliferation of osteoblasts [139]. In addition, enhanced WTAP activity can lead to m6A methylation and degradation of *HMBOX1* mRNA, an activator of PI3K-AKT pathway, which also in turn inhibits excessive proliferation of osteoblasts towards osteosarcoma [140]. Another study showed that repression of *Fto* by *miR-22-3p* leads to elevation of m6A methylation on *c-Myc* mRNA and its degradation and subsequently enhanced osteoblast differentiation. Since c-Myc acts as an upstream activator of PI3K, its negative effects on osteoblast differentiation is mediated by activation of PI3K-AKT pathway [141]. In contrast, an earlier study showed that during osteogenic differentiation, the expression level of METTL3 is significantly increased leading to enhanced phosphorylation of AKT and in turn activating PI3K-AKT signaling pathway [142]. Similarly, during periodontitis, increased *Mettl3* expression caused osteogenic differentiation of periodontal ligament stem cells (PDLSCs) through activation of PI3K-AKT signaling pathway [143, 144]. Consistent with this finding, another study later demonstrated ALKBH5 eraser negatively regulates the osteogenic differentiation through m6A-demethylation and mRNA degradation of *PRMT6*, an activator of PI3K-AKT signaling pathway, which in turn impairs osteoblast differentiation [145]. Yet, during the ossification of the ligamentum flavum, ALKBH5 appeared to play opposite role by enhancing osteogenic differentiation through m6A-dependent phosphorylation of AKT and activation of the AKT signaling pathway [47]. However, the detail mechanism by which ALKBH5 increases AKT phosphorylation remained unknown. These findings might indicate that the m6A-mediated regulation of PI3K-AKT signaling can have either positive or negative effects on osteoblastogenesis depending on the interplay of upstream mediators of these regulatory effects. During osteoclastogenesis, Traf6, a major adaptor protein recruited by the Rankl-Rank interaction, initiates the activation of PI3K-AKT signaling to promote osteoclast differentiation. Interestingly, the knockdown of *Mettl3* causes the retention of *Traf6* in the nucleus and its decreased translation which in turn impairs osteoclast differentiation and bone resorption [32]. Similarly, *Ythdf1* knockdown decreased *Traf6* mRNA stability, and thereby inactivated PI3K-AKT signaling and impaired osteoclast differentiation [128].

Compared to bone, fewer studies are conducted in other skeletal tissues investigating regulatory connection between PI3K-AKT signaling and m6A RNA modifications. In developing tooth, for instance, Alkbh5 enhances phosphorylation and activity of PI3K-AKT pathway to promote dental papilla cell line odontoblastic differentiation [146]. In cartilage, a lncRNA (*OANCT*) can bind to FTO and reduce the m6A demethylation of *PIK3R5* mRNA by FTO, and in turn promotes *PIK3R5* mRNA stability. The maintained stability of *PIK3R5* keeps the PI3K-AKT signaling in activated mode accelerating chondrocyte apoptosis and osteoarthritis [147]. During inflammatory degeneration of cartilage, METTL3 promotes the maturation of *miR-126-5p* which inhibits the PI3K-AKT pathway by down-regulating *PIK3R2* expression causing chondrocyte apoptosis [148].

### Hippo Signaling Pathway

The Hippo signaling pathway, recognized as a growth signaling pathway, utilizes YAP/TAZ as key effectors to regulate organ growth during development, influencing tissue homeostasis [149]. YAP/TAZ act as transcriptional co-factors, interacting with various transcription factors and signaling pathways to modulate gene expression related to proliferation, growth, apoptosis, and differentiation. Skeletal cell differentiation is governed by YAP/TAZ, directing mesenchymal stem cell commitment towards osteoblastic lineage while inhibiting adipogenesis and chondrogenesis [150, 151]. Recent studies suggest that YAP/TAZ's impact on bone development varies, affecting osteoblastogenesis, bone formation, and osteoclast activity [152]. In chondrogenesis, YAP induces chondrocyte proliferation while inhibiting differentiation, primarily through Sox6 expression and BMP response control [150]. While YAP/TAZ primarily localize in the nucleus of pre-hypertrophic chondrocytes, promoting chondrocyte commitment while blocking hypertrophic differentiation, TAZ promotes chondroprogenitor cell proliferation while inhibiting chondrocyte maturation. Regarding skeletal tissue development and morphogenesis, recent investigations suggest that YAP/TAZ primarily control skeletal morphology, with their absence resulting in abnormal skeletal elements and cleft palate, while their overactivation leads to severe skeletal malformations, indicating their crucial role in skeletal tissue morphogenesis [153].

Current evidence for a direct regulatory link between the Hippo signaling pathway and m6A RNA modification remains scant. Recent findings have identified CHST11, a chondrogenic factor implicated in osteoarthritis, as a target for m6A methylation by a novel writer, KIAA1429. This modification leads to reduced *CHST11* mRNA stability upon recognition by the YTHDF2 reader [154, 155]. Intriguingly, CHST11 has been recognized as a direct interactor with the Hippo pathway, enhancing *MOB1B* expression and thereby activating the Hippo–YAP signaling [155]. However, the direct regulatory impact of this interaction during chondrogenesis requires further investigation. Another study highlighted that m6A-methylated YAP transcripts, when recognized by YTHDF1, facilitate its translation, enhancing osteosarcoma cell proliferation [156]. This research also discovered that ALKBH5-dependent m6A demethylation of RNAs significantly hinders the growth and mobility of osteosarcoma cells by directly and indirectly regulating Hippo-YAP signaling. Indirectly, ALKBH5-mediated m6A demethylation promotes the maturation of *pre-miR-181b-1*, which subsequently inhibits YAP and its proliferative effects. Directly, ALKBH5 inhibits m6A methylation of *YAP*, suppressing its mRNA stability and translation [156]. Yet, the validation of these direct and indirect regulatory mechanisms during bone formation under non-pathological conditions is still pending. It should be noted that a recent study has revealed that METTL3 expression in bone leads to m6A methylation-dependent increase in stability of *RASSF1* mRNA (a component of Hippo pathway and an inducer of osteoblastogenesis), and thus stimulates osteoblast proliferation [157].

## Regulatory Connections Between m6A RNA Methylation and Signals Mediated by Nuclear Receptors

**Fig. 5:**
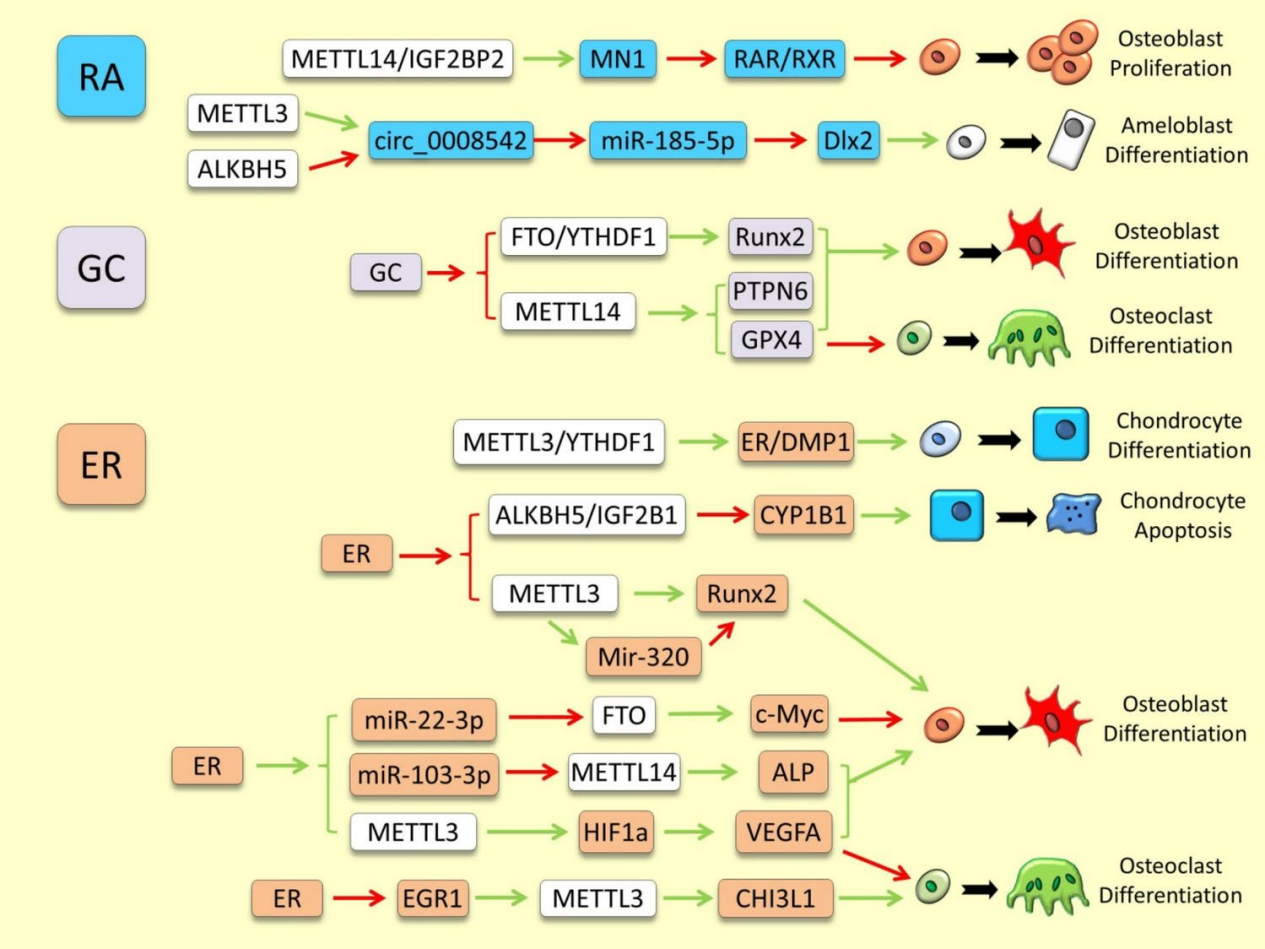
Examples of m6A RNA methylation dependent regulation of nuclear receptor signaling pathways in skeletal tissues. The green and red arrows indicate induction/enhancement and inhibition/degradation, respectively

The most notable regulatory interactions between m6A RNA methylation modifiers and the pathway components presented in this section are depicted in Fig. 5, and are also outlined in Supplementary Table 3.

### Retinoic Acid Signaling Pathway

Retinoic Acid (RA), one of the earliest identified vertebrate morphogens [158], plays critical roles in developmental patterning. Derived from its inactive precursor, Vitamin A (retinol), RA is essential for growth, development, and tissue maintenance [159, 160]. RA diffuses rapidly and activates specific nuclear receptors, primarily heterodimers of RXRα and RAR (α, β, and γ), to regulate RA-responsive gene expression through unique DNA sequences. In vertebrates, RA signaling governs early skeletal morphogenesis and anterior–posterior embryonic patterning by modulating homeobox gene expression [158]. Dysregulation of RA synthesis or signaling, including mutations in RA receptor genes and enzymes such as Rdh10 and Raldh3, is linked to skeletal abnormalities [161–164]. Enzymes responsible for RA metabolism regulate spatiotemporal RA levels after its synthesis, further influencing skeletal development.

To date only one study has investigated a direct regulatory connection between m6A RNA modification and RA signaling in skeletal tissues. In that study, it has been found that METTL14 contributes to osteosarcoma progression by m6A methylation and increasing the mRNA stability of *MN1* (guided by IGF2BP2 reader), which is a potent inhibitor of RAR/RXR-mediated transcription. The MN1 interference in RAR/RXR transcription causes excessive proliferation of osteoblasts while inhibiting their terminal differentiation [119]. During tooth/jaw development, RA signal enhances ameloblast and osteoblast differentiation through the suppression of *miR-185-5p*, an inhibitor of *Dlx2* which is required for developmental amelogenesis and osteogenesis [165]. Interestingly, both METTL3 and ALKBH5 can indirectly act at upstream of *miR-185-5p* (in opposite manner) through affecting mRNA stability of *circ_0008542*; an inhibitor of miR-185-5p during bone formation and resorption [166]. Therefore, even though the regulatory connection between RA signal and METTL3 or ALKBH5 through expression regulation of *miR-185-5p* is a likely scenario but this has not been investigated in any skeletal tissues.

### Glucocorticoid Signaling Pathway

Glucocorticoids (GCs), derived from steroids, bind glucocorticoid receptors (GR) present in nearly all tissues [167]. GCs cross cell membranes and modulate transcription via nuclear GR, activated upon ligand binding. Ligand-bound GR regulates gene transcription positively or negatively through interactions with other TFs. GR signaling plays a role in skeletogenesis and morphological adaptation of skeletal structures [168, 169]. GC-induced osteoporosis arises from GR pathway interactions with skeletal cell regulatory signals [169]. GR signaling elements respond to environmental and cellular stresses [170]. Maternal GR transcripts are critical for early skeletal development in zebrafish embryos [171]. Elevated GC levels during growth subtly impact craniofacial and vertebral structures and cause skeletal pathologies in adulthood in (e.g., in humans and fish [172–175]). Genes involved in ECM biogenesis are direct GR pathway effectors during skeletogenesis [173, 176].

It has been recently shown that activation of GC inhibits *Fto* transcription which leads to reduced mRNA stability of osteoblast markers such as *Alpl*, *Col1a1* and *Runx2* and in turn impairs osteoblast differentiation. These effects can be reversed by overexpression of *Fto* through Ythdf1-guided increased mRNA stability of the target genes [177]. This demonstrates that GC signal can act as upstream inhibitor of m6A RNA methylation process during osteoblastogenesis. On the other hand Mettl14 inhibits osteoclast differentiation by increasing m6A mediated stability of *Gpx4*, thus enhancing bone formation versus bone resorption [178]. Another study also showed that GC-induced osteonecrosis is caused by reduced Mettl14 and m6A methylation level accompanied by decreased mRNA stability of *PTPN6*, a stimulator of osteoblast proliferation and differentiation [92]. Importantly, *GPX4* is a major downstream effector of GC signaling pathway, and during GC-induced osteoporosis, GC mediates its osteoclastogenesis effects by inhibiting *GPX4* expression [179]. These findings indicate opposing role of GC signal and Mettl14-dependent m6A methylation during osteoclast differentiation. Yet, such opposing regulatory connections between m6A RNA modification and GC signaling in cartilage and tooth remained to be elucidated.

### Estrogen Signaling Pathway

Estrogens, hormones derived from androgenic precursor molecules, were originally identified as sex hormones but are now known to influence various developmental and physiological processes, including skeletal system formation and regeneration [180, 181]. This role aligns with the prevalence of sexual dimorphism, largely driven by sex-hormone signaling. In skeletal cells, estrogens signal through two receptor types: ligand-regulated estrogen receptors (ER-alpha/-beta) [180] and G-protein-associated receptors (e.g., GPR-30 and GPER1) [182–184]. These estrogen signaling mediators, present in chondrocytes, play crucial roles in chondrogenesis [184, 185]. Estrogen's effects on chondrocyte proliferation and cartilage development differ among species [186–188]. Elevated estrogen levels during zebrafish development can severely disrupt craniofacial and trunk skeletal formation [186–189].

Premature closure of the growth plate in long bones, induced by estrogen, significantly contributes to short stature following early puberty. In this phenomenon, chondrocytes can directly transform into osteoblasts as part of endochondral ossification, a process referred to as chondrocyte osteogenesis. Recent research has identified *DMP1*, a direct downstream target of ERα/β, to play an essential role in the estrogen-mediated regulation of chondrocyte osteogenesis [190]. Interestingly, another study demonstrated that METTL3-dependent m6A modification regulates hypertrophic differentiation of chondrocytes through YTHDF1-guided enhancement of *DMP1* mRNA stability which leads to endochondral ossification [191]. During osteoarthritis progression m6A might act at upstream of estrogen signal by regulating CYP1B1, a major enzyme in estrogen metabolism. In this study, it was found that osteoarthritis downregulates ALKBH5 eraser and increases *CYP1B1* mRNA stability (guided by IGF2BP1 reader), thereby enhancing mesenchymal stem cell (MSC) senescence which are precursor cells differentiating to both osteoblasts and chondroblasts (impaired cartilage and bone formation) [192].

In bone, the estrogen-deficiency induced osteoporosis is the result of *Mettl3* downregulation which leads to decreased mRNA stability of *Runx2* and therefore impaired osteoblast differentiation. Interestingly, the same study also found an indirect mechanism with similar results through which the estrogen-dependent downregulation of Mettl3 caused enhanced expression *mir-320* which in turn targets *Runx2* and inhibits its translation [193]. The METTL3 mediated inhibition of *mir-320* was proposed to be guided by YTHDF2 which recognizes m6A methylation of *mir-320* and leads to its decay. Later it has been found that an activator of ERα/β increases METTL3 expression in bone leading to m6A methylation-dependent increase in stability of *HIF-1α* and *VEGF-A* mRNAs and stimulation of osteoblast differentiation (while inhibiting osteoclast differentiation) [157]. Interestingly, estrogen has been found to regulate osteoclastogenesis through an indirect and hierarchical regulatory connection with METTL3. In this process, estrogen deficiency causes upregulation of EGR1 which promotes METTL3 transcription and increases m6A-dependent *CHI3L1* mRNA stability (stimulator of *NAFTc1* expression), thereby stimulating osteoclast differentiation [194]. These findings suggest that estrogen signal can regulate both osteoblast and osteoclast differentiations respectively through direct and indirect regulation of *METTL3* expression. An alternative scenario has been also proposed in estrogen-deficiency induced osteoporosis; i.e., reduced estrogen level decreases the expression of *miR-103-3p* and subsequently increased expression of its direct target, *METTL14*, as well as m6A methylation of osteogenic markers (*Alp, Bglap*, and *Col1α1*) [195]. The m6A methylation of these markers accompanied with their increase expression (probably via enhancement of their mRNA stability), and thereby stimulation of osteoblast proliferation, differentiation, and matrix mineralization [195]. However, the estrogen/*miR-103-3p*/*METTL14* axis appeared to have no effect on osteoclasts indicating that this mechanism do not explain the estrogen mediated balance between osteoblast and osteoclast differentiations. Finally, *miR-22-3p*, an estrogen-induced microRNA, is a direct negative regulator of *FTO* expression in osteoblasts, and its repressive effects on FTO causes increased m6A-dependent degradation of *c-Myc* mRNA which subsequently promotes osteoblast differentiation [141].

## Regulatory Connections Between m6A RNA Methylation and Calcium Dependent Pathways

**Fig. 6:**
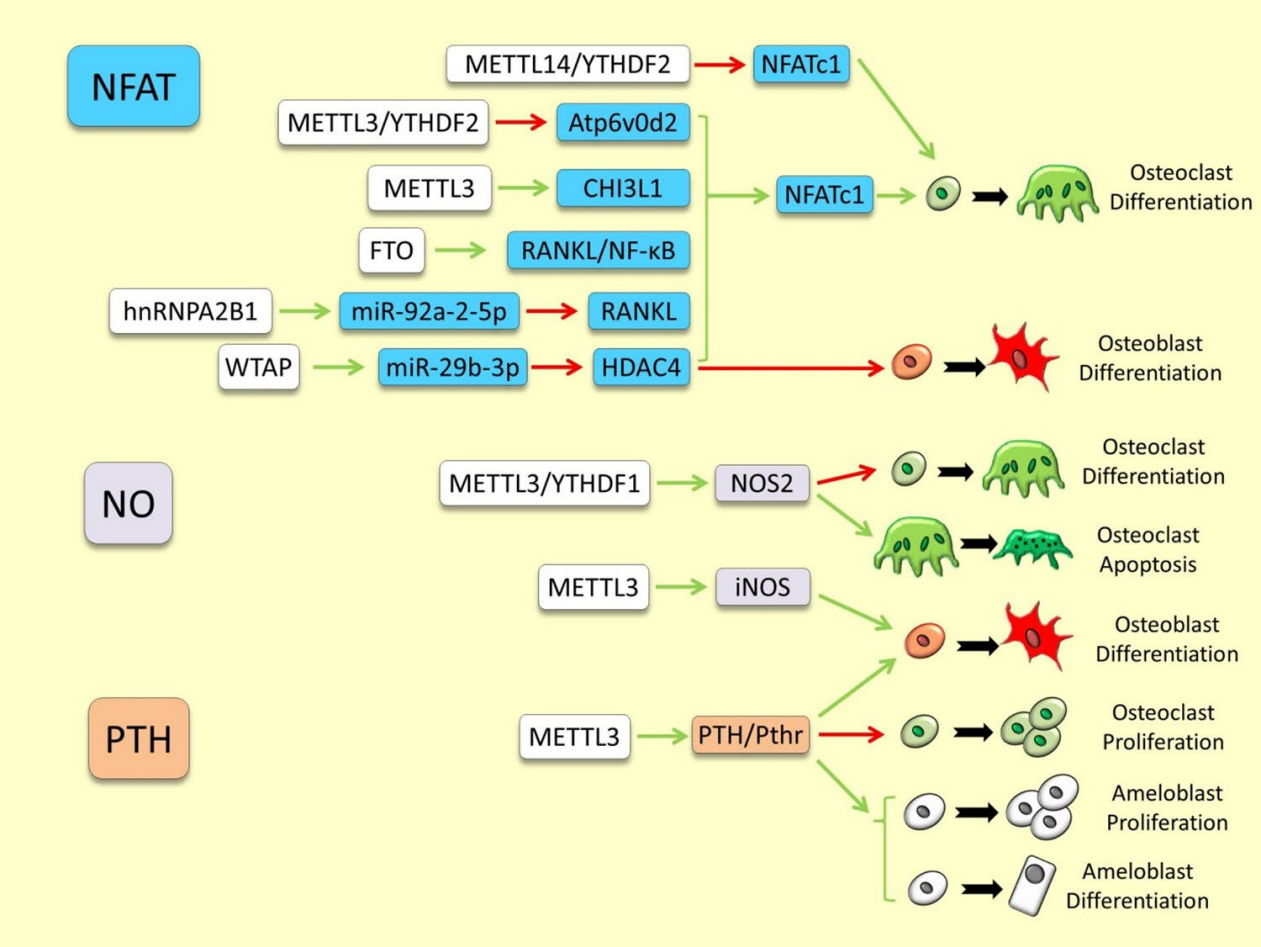
Examples of m6A RNA methylation dependent regulation of calcium mediated signaling pathways in skeletal tissues. The green and red arrows indicate induction/enhancement and inhibition/degradation, respectively

The most notable regulatory interactions between m6A RNA methylation modifiers and the pathway components presented in this section are depicted in Fig. 6, and are also outlined in Supplementary Table 4.

### NFAT Signaling Pathway

The NFAT (nuclear factor of activated T-cells) signaling pathway plays a crucial role in skeletal development and morphogenesis, influencing osteoblastogenesis, osteoclastogenesis, skeletal remodeling, inflammation, and homeostasis through calcium-dependent transduction of extracellular cues into gene expression changes essential for bone formation and maintenance [196, 197]. Activation of NFAT, particularly NFATc1, a primary downstream transcription factor, promotes mesenchymal stem cell differentiation into osteoblasts by upregulating osteogenic genes like Runx2 and Osterix [198]. NFAT also regulates osteoclast differentiation by inducing genes vital for osteoclast activity, such as TRAP and CTSK, thereby maintaining osteoblast-osteoclast balance and bone remodeling [199]. Dysregulation of NFATc1 can lead to bone pathologies like osteoporosis and osteopetrosis [196, 200]. Its evolutionary conservation across vertebrates highlights its significance in comparative bone remodeling studies [201]. Moreover, NFATc1 modulates bone and cartilage inflammation by activating pro-inflammatory cytokines in immune cells and maintains skeletal homeostasis by controlling genes related to bone metabolism, mineralization, and turnover, ensuring proper skeletal health throughout life [199, 202, 203].

An increasing number of studies have revealed extensive and complex regulatory connections between NFAT signaling and m6A RNA modification in skeletal tissues particularly in modulation of osteoclastogenesis. During bone remodeling, NFATc1 directly binds to the promoter of *Atp6v0d2* and induces its expression leading to differentiation of pre-osteoclasts [31]. On the other hand, *Atp6v0d2* has been found to be degraded by METTL3-dependent m6A methylation (guided by YTHDF2) which impaired osteoclast function [32]. These observations proposed opposing regulatory roles of METTL3 dependent m6A methylation and NAFTc1 mediated signal on *Atp6v0d2* in different stages of osteoclastogenesis and bone remodeling. However, a later study has suggested that METTL3 can also act upstream of NFATc1 by enhancing m6A methylation and mRNA stability of *CHI3L1*, which in turn upregulates *NAFTc1* expression and promotes osteoclast differentiation [204]. An indirect positive regulation of NFATc1 by METTL3/m6A-dependent mechanism has been reported in inflammatory bone through repression of nitric oxide (NO) pathway [205]. In this study, METTL3-dependent m6A methylation of NOS2 mRNA decreased its stability and blocked NO signaling which in turn induced NFATc1 and osteoclast differentiation. The METTL14-dependent m6A methylation of NFATc1 has been found to inhibit osteoclast differentiation by decreasing *NFATc1* mRNA stability after YTHDF2 recognition [206]. Conversely, FTO mediated m6A demethylation of NFATc1 has induced osteoclast differentiation probably though increasing its mRNA stability [207]. Interestingly, the osteoclastogenic effect of FTO on NFATc1 can be mediated through an indirect mechanism as well; as FTO expression facilitates RANKL-induced binding of NF-κB to NFATc1 promoter and then promoted osteoclast differentiation and bone resorption [208]. Such an indirect regulatory connection has been proposed for METTL14/m6A dependent increased mRNA stability of *GPX4*, which inhibits RANKL-induced NFATc1 activity and impaired osteoclast differentiation [178]. These findings represent opposite regulatory roles of METTL14 and FTO in both direct and indirect regulation of NFATc1 activity during osteoclastogenesis. The other m6A writer, WTAP, also promotes osteogenesis through m6A mediated maturation of *miR-29b-3p* which targets *HDAC4* and inhibits NFATc1 function in osteoclast differentiation [209]. The NFATc1 function can also be regulated by m6A readers during skeletogenesis. For instance, NFATc1 function is blocked by YTHDC1/m6A dependent enhancement *PTPN6* mRNA stability which is an upstream inhibitor of NFATc1 during osteoclastogenesis [210]. In addition, YTHDF2 has a negative regulatory role in LPS-induced osteoclast differentiation and the inflammatory response via m6A mediated degradation of Nfatc1 mRNA [127]. A less studied reader, hnRNPA2B1, also was found to be involved in skeletogenesis through exosomal upregulation of *miR-92a-2-5p* which represses *IRF8*, and consequently, activation of RANKL-induced *NFATc1* expression, osteoclastogenesis and bone resorption [211].

### Nitric Oxide Signaling Pathway

The Nitric oxide (NO) signaling pathway relies on calcium ions for the activation of nitric oxide synthase enzymes, with calcium influx triggering nitric oxide production, which in turn regulates various physiological processes. NO signaling was first identified as a regulator of endochondral ossification and later recognized for its role in skeletal cell differentiation and mechanical adaptation [212–214]. Currently, only two studies have explored the direct regulatory relationship between m6A RNA modification and the nitric oxide signaling pathway in skeletal tissues [205, 215]. The METTL3 expression is decreased during inflammatory induction of osteoclastogenesis and the reduction in activity of METTL3 increases the stability of *NOS2* mRNA and *iNOS* (inducible NOS) protein (through YTHDF1-dependent manner). Consequently, activated NO signal inhibits osteoclast differentiation and promotes their apoptosis [205]. Moreover, METTL3 plays a key role in macrophage polarization, a process required for controlling the osteogenic differentiation and migration during bone regeneration. Interestingly, this process is mediated by METTL3-dependent m6A methylation and increased mRNA stability of *iNOS* leading to macrophage-induced osteoblast differentiation and bone formation [215].

### PTH Signaling Pathway

Parathyroid hormone (PTH) and parathyroid hormone-related peptide (PTHrP) are closely related proteins secreted by different cell types. PTH, produced by the parathyroid glands, maintains calcium and phosphate levels in the blood, while PTHrP, with variations due to RNA splicing, functions as a paracrine/autocrine hormone involved in growth and maturation [216]. These proteins bind to distinct or overlapping receptors, activating various signaling pathways, including Ca2 + elevation, enzyme activation (e.g., PKA and PLC), and modulation of pathways like MAPK [216]. The PTH/PTHrP pathways regulate osteoblastogenesis and bone formation [217, 218]. PTHrP signaling affects SOX9 and RUNX2, key regulators of cartilage and bone formation, and modulates RANKL and AP-1 activity in skeletal cells.

To date, there are only two studies in tooth and bone investigated the direct regulatory connection between m6A RNA modifiers and PTH signaling pathway [219, 220]. In mice, a conditional knockout study of *Mettl3* showed the development osteoporosis-like symptoms, characterized by diminished bone formation and reduced osteogenic differentiation [219]. The study reveals the Pth/Pth1r signaling axis as a key downstream pathway affected by m6A regulation, where Mettl3 deletion decreases Pth1r translation efficiency and disrupts Pth-driven osteogenic responses. The activated Mettl3/Pth-Pth1r axis accompanied with increased level of osteoblast proliferation and differentiation whereas osteoclast had decreased proliferation leading to promotion in bone formation [219]. Similarly, during tooth development depletion of *Mettl3* caused reduced translation of Pthr1 and impaired odontoblast proliferation, migration and differentiation [220].

## Conclusion

The numerous and complex crosstalk between m6A RNA methylation and skeletogenic signaling pathways reveals a sophisticated layer of post-transcriptional regulation influencing skeletal development, homeostasis, and disease. This review demonstrates that m6A RNA methylation modulates key pathways, and other less studied skeletogenic pathways, to orchestrate cellular processes such as osteoblast and osteoclast differentiation, chondrocyte maturation, and bone remodeling. The dual roles of m6A modifiers—such as METTL3, METTL14, ALKBH5, and FTO—in both promoting and inhibiting specific signaling cascades underscore their dynamic regulatory potential. These findings also emphasize the tissue-specific and context-dependent nature of m6A's impact, suggesting a balance between methylation and demethylation that finely tunes skeletal tissue development and regeneration. Importantly, the connection between m6A methylation and signaling pathways in both normal and pathological skeletal tissues highlights its role not only in maintaining homeostasis but also in driving disease processes such as osteosarcoma, osteoporosis, and osteoarthritis. Despite significant progress, several questions remain unanswered, including the precise upstream regulators of m6A dynamics, the interplay between m6A and non-coding RNAs in skeletal biology, and how environmental and mechanical factors influence these regulatory networks. Future research should aim to delineate the context-specific roles of m6A modifiers and readers in distinct skeletal cell types and developmental stages. Advanced technologies like single-cell transcriptomics and epitranscriptomic profiling will be critical for uncovering the spatial and temporal dynamics of m6A modifications. Moreover, translating these insights into therapeutic interventions, such as targeted modulation of m6A pathways, holds promise for treating skeletal disorders and enhancing tissue regeneration. As our understanding of m6A-mediated regulation expands, it is poised to reshape the field of skeletal biology and regenerative medicine.

## Supplementary Information

Below is the link to the electronic supplementary material.

Supplementary file1 (DOCX 22 KB)

## Abbreviations

ACLY: ATP Citrate Lyase
AKT: Protein Kinase B (PKB)
ALKBH5: AlkB Homolog 5, RNA Demethylase
Alpl: Alkaline Phosphatase, Liver/Bone/Kidney
AP-1: Activator Protein-1
Atp6v0d2: ATPase H + Transporting V0 Subunit D2
Beclin-1: Autophagy-related Protein Beclin-1
Bglap: Bone Gamma-Carboxyglutamate Protein (Osteocalcin)
BMPR: Bone Morphogenetic Protein Receptor
BMPs: Bone Morphogenetic Proteins
c-Fos: Proto-Oncogene c-Fos
CHI3L1: Chitinase-3-Like Protein 1
CHST11: Carbohydrate Sulfotransferase 11
circRERE: Circular RNA RERE
c-Jun: Proto-Oncogene c-Jun
c-Myc: Proto-Oncogene c-Myc
Col1a1: Collagen Type I Alpha 1 Chain
CtsK: Cathepsin K
CYP1B1: Cytochrome P450 Family 1 Subfamily B Member 1
DAC1: DNA Cytosine-5-Methyltransferase 1
Dhh: Desert Hedgehog
Dixdc1: Disheveled Binding Antagonist of Beta-Catenin 1
Dkks: Dickkopf-Related Proteins
Dll: Delta-Like Ligands (Notch Signaling)
Dlx2: Distal-Less Homeobox 2
DMP1: Dentin Matrix Protein 1
DNMT1: DNA (Cytosine-5)-Methyltransferase 1
DPSCs: Dental Pulp Stem Cells
ECM: Extracellular Matrix
EGF: Epidermal Growth Factor
EGR1: Early Growth Response 1
ER: Estrogen Receptor
ERK: Extracellular Signal-Regulated Kinases
Fem1b: Feminization 1 Homolog B
FGFR: Fibroblast Growth Factor Receptor
FGFs: Fibroblast Growth Factors
FRZB: Frizzled-Related Protein (Secreted)
FTO: Fat Mass and Obesity-Associated Protein (m6A Demethylase)
FZD: Frizzled Receptors (Wnt Signaling)
GAS5: Growth Arrest-Specific 5 (Long Non-Coding RNA)
GCs: Glucocorticoids
GDF11: Growth Differentiation Factor 11
Gli: GLI-Kruppel Family Zinc Finger Proteins (Hedgehog Signaling)
GPER1: G Protein-Coupled Estrogen Receptor 1
GPR-30: G Protein-Coupled Receptor 30
GPX4: Glutathione Peroxidase 4
GR: Glucocorticoid Receptor
GSK-3β: Glycogen Synthase Kinase-3 Beta
HDAC4: Histone Deacetylase 4
HES1: Hairy and Enhancer of Split-1
HEY: Hairy/Enhancer-of-split Related with YRPW Motif
Hh: Hedgehog Signaling Pathway
HIF-1α: Hypoxia-Inducible Factor 1-Alpha
HMBOX1: Homeobox Containing 1
IGF2BP: Insulin-Like Growth Factor 2 mRNA Binding Proteins
IGFs: Insulin-Like Growth Factors
Ihh: Indian Hedgehog
iNOS: Inducible Nitric Oxide Synthase
IRF2BPL: Interferon Regulatory Factor 2 Binding Protein-Like
JNK: C-Jun N-terminal Kinase
Kremen1/2: Kringle Containing Transmembrane Proteins 1 and 2
Lef: Lymphoid Enhancer-Binding Factor
lncSNHG7: Long Non-Coding RNA Small Nucleolar RNA Host Gene 7
LPS: Lipopolysaccharides
LRPs: Low-Density Lipoprotein Receptor-Related Proteins
LTBP: Latent Transforming Growth Factor Beta Binding Protein
m6A: N6-Methyladenosine
MAPK: Mitogen-Activated Protein Kinase
METTL14: Methyltransferase-Like 14 (m6A Methylation)
METTL16: Methyltransferase-Like 16
METTL3: Methyltransferase-Like 3 (m6A Writer)
METTL5: Methyltransferase-Like 5
MN1: Meningioma 1
MOB1B: MOB Kinase Activator 1B
MSCs: Mesenchymal Stem Cells
mTOR: Mechanistic Target of Rapamycin
NELL1: Neural EGFL-Like 1
NFAT: Nuclear Factor of Activated T Cells
NFATc1: Nuclear Factor of Activated T-Cells, Cytoplasmic 1
NO: Nitric Oxide
NOG: Noggin (BMP Inhibitor)
Notch: Notch Signaling Pathway
OANCT: Osteoarthritis and New Cartilage Tissue
OPG: Osteoprotegerin
p38: P38 Mitogen-Activated Protein Kinase
PDLSCs: Periodontal Ligament Stem Cells
PDPK1: 3-Phosphoinositide Dependent Protein Kinase 1
PI3K: Phosphoinositide 3-Kinase
PIK3R5: Phosphoinositide-3-Kinase Regulatory Subunit 5
PKA: Protein Kinase A
PLC: Phospholipase C
PRMT6: Protein Arginine Methyltransferase 6
Ptch: Patched (Hedgehog Signaling Receptor)
PTH: Parathyroid Hormone
Pth1r: Parathyroid Hormone 1 Receptor
PTHrP: Parathyroid Hormone-Related Protein
PTPN6: Protein Tyrosine Phosphatase Non-Receptor Type 6
RA: Retinoic Acid
Raldh3: Retinal Dehydrogenase 3
RANK: Receptor Activator of Nuclear Factor Kappa-Β
RANKL: Receptor Activator of Nuclear Factor Kappa-B Ligand
RAR: Retinoic Acid Receptor
RASSF1: Ras Association Domain Family Member 1
RBM15: RNA Binding Motif Protein 15
RBPjk: Recombination Signal Binding Protein for Immunoglobulin Kappa J Region
Rdh10: Retinol Dehydrogenase 10
Ror2: Receptor Tyrosine Kinase-Like Orphan Receptor 2
RP11-44: Long Non-Coding RNA RP11-44
RUNX2: Runt-Related Transcription Factor 2
RXR: Retinoid X Receptor
Ryk: Receptor-Like Tyrosine Kinase
Sfrps: Secreted Frizzled-Related Proteins
Shh: Sonic Hedgehog
Sirt1: Sirtuin 1
SKP2: S-Phase Kinase-Associated Protein 2
SLC25A1: Solute Carrier Family 25 Member 1
Smad: SMAD Family Transcription Factors (TGF-β Signaling)
Smo: Smoothened (Hedgehog Signaling)
Sost: Sclerostin
SOSTDC1: Sclerostin Domain Containing 1
Sox9: SRY-Box Transcription Factor 9
SRF: Serum Response Factor
TAZ: Transcriptional Coactivator with PDZ-Binding Motif (WWTR1)
TCFs: T-Cell Factor Family (Wnt Signaling)
TGF-β: Transforming Growth Factor Beta
Traf6: TNF Receptor-Associated Factor 6
TRAP: Tartrate-Resistant Acid Phosphatase
TRMT112: TRNA Methyltransferase Subunit 112
TWIST1: Twist Family BHLH Transcription Factor 1
VEGF-A: Vascular Endothelial Growth Factor A
WGD: Whole Genome Duplication
Wif1: Wnt Inhibitory Factor 1
Wnt: Wingless-Related Integration Site (Wnt Signaling Pathway)
WTAP: Wilms Tumor 1-Associating Protein (m6A Methylation)
YAP: Yes-Associated Protein (Hippo Signaling)
YTHDF: YT521-B Homology Domain Family (m6A Readers)
ZC3H13: Zinc Finger CCCH-Type Containing 13
