# Supplementary material for: Regulation of Skeletogenic Pathways by m6A RNA Modification: A Comprehensive Review
Source: Calcif Tissue Int. 2025 Apr 3;116(1):58. doi: 10.1007/s00223-025-01367-9 (PMC11968561; doi:10.1007/s00223-025-01367-9)
Supplement: Supplementary file 1 — Supplementary file1 (DOCX 22 KB) [file 223_2025_1367_MOESM1_ESM.docx]

| **Pathway** | **m6A RNA Component** | **Pathway Component** | **Target Tissue** | **Cellular Outcome** | **Reference** |
| --- | --- | --- | --- | --- | --- |
| TGF-β | FTO | GDF11 | Bone | Inhibits osteoblast differentiation and bone formation | Shen et al., 2018 |
|  | YTHDF3 | GAS5 | Bone | Regulates TGF-β signal with roles in bone pathobiology | Zhou et al., 2022 |
|  | METTL3 | miR-196b-5p | Bone, Tooth | Enhances osteo/odontogenic differentiation | Han et al., 2023 |
|  | METTL3 | Atp6v0d2 | Bone | Impairment of osteoclast function and bone remodeling | Li et al., 2020 |
| BMP | METTL3 | BMP2 | Bone | Promotes osteogenic differentiation | Liu et al., 2021 |
|  | METTL3 | LINC00657 | Bone | Enhances osteoblast differentiation | Peng et al., 2022 |
|  | ALKBH5 | BMP2 | Bone | Enhances ossification | H. F. Wang et al., 2020 |
| Hedgehog | METTL3 | PTCH1, GLI2 | Nervous system | Regulates RNA stability and translation | Zhang et al., 2022 |
|  | WTAP | SHH | Tooth | Enhances ameloblast differentiation and enamel formation | Xie et al., 2022 |
| Wnt | METTL3 | LEF1 | Bone | Activates Wnt/β-catenin signaling and promotes osteoblast differentiation | Miao et al., 2019 |
|  | FTO | DAC1 | Bone (osteosarcoma cells) | Accelerates proliferation by reducing DAC1 mRNA stability | D. Lv et al., 2022 |
|  | WTAP | miR-181a/c | Bone | Promotes osteogenic differentiation by suppressing SFRP1 | You et al., 2023 |
| Notch | METTL3 | Notch1 | Skeletal tissue | Inhibits Notch pathway, important for angiogenesis | Yao et al., 2024 |
|  | FTO | Notch signaling components | Bone (under mechanical stress) | Elevates expression of Notch pathway components, promoting osteogenic differentiation | Sun et al., 2022 |
|  | ALKBH5 | Notch1, Notch2 | Osteosarcoma cells | Suppresses growth, migration, and invasion by enhancing RNA stability of Notch1 and Notch2 | D. Chen et al., 2023 |
|  | KIAA1429 | miR-143-3p, Notch1 | Osteosarcoma cells | Represses miR-143-3p, increases Notch1 expression, promoting proliferation, migration, and invasion | Han et al., 2020 |

**Table 1. Examples of known regulatory connections between components of m6A RNA methylation and major skeletal development pathways.**

**Table 2. Examples of known regulatory connections between components of m6A RNA methylation and growth factors mediated signals in skeletal system.**

| **Pathway** | **m6A RNA Component** | **Pathway Component** | **Target Tissue** | **Cellular Outcome** | **Reference** |
| --- | --- | --- | --- | --- | --- |
| Growth factors | ALKBH5 | IGF-2 | Bone | Promotes osteogenesis and ossification | H.-F. Wang et al., 2020 |
|  | METTL3 | miR-7212-5p | Bone | Inhibits osteoblast differentiation by targeting FGFR3 | Mi et al., 2020 |
|  | METTL3 | ACLY, SLC25A1 | Dental pulp stem cells | Induces odontoblastic differentiation by increasing mRNA stability | Cai et al., 2022 |
|  | IGF2BP2 | SRF | Bone | Enhances osteoblast proliferation and osteogenesis by stabilizing SRF mRNA | Z. Zhou et al., 2023 |
|  | METTL14 | Beclin-1 | Bone | Enhances osteoblast differentiation and suppresses osteoclastogenesis | Mingyu He et al., 2022 |
| MAPK | METTL3 | NFATc1 | Bone | Enhances MAPK signaling promoting osteoclast differentiation and bone resorption | Li et al., 2020 |
|  | YTHDF2 | MAPKs (Map4k4, Map2k3, Map2k4) | Bone | Negatively regulates osteoclastogenesis and inflammatory response | Fang et al., 2021 |
|  | METTL3 | circRNA3634 | Cartilage | Enhances chondrocyte proliferation, differentiation, and migration via MAPK1 activation | Song et al., 2023 |
| PI3K-AKT | WTAP | HMBOX1 | Bone | Inhibits excessive osteoblast proliferation by degrading HMBOX1 mRNA | Chen et al., 2020 |
|  | FTO | c-Myc | Bone | Enhances osteoblast differentiation by degrading c-Myc mRNA | Zhang et al., 2020 |
|  | ALKBH5 | PRMT6 | Bone | Impairs osteoblast differentiation by degrading PRMT6 mRNA | Li et al., 2021 |
| Hippo | KIAA1429 | CHST11 | Cartilage | Reduces CHST11 mRNA stability, activating Hippo-YAP signaling | X. Chen et al., 2023 |
|  | ALKBH5 | YAP | Bone | Suppresses YAP mRNA stability and translation, hindering cell growth | Yuan et al., 2021 |
|  | METTL3 | RASSF1 | Bone | Stimulates osteoblast proliferation by stabilizing RASSF1 mRNA | Tian et al., 2023 |

**Table 3. Examples of known regulatory connections between components of m6A RNA methylation and signals mediated by nuclear receptors in skeletal system.**

| **Pathway** | **m6A RNA Component** | **Pathway Component** | **Target Tissue** | **Cellular Outcome** | **Reference** |
| --- | --- | --- | --- | --- | --- |
| RA | METTL14 | MN1 | Bone | Promotes osteosarcoma progression and inhibits osteoblast terminal differentiation | Mingyu He et al., 2022 |
|  | METTL3, ALKBH5 | circ_0008542 | Bone, Tooth | Regulates miR-185-5p to enhance ameloblast and osteoblast differentiation | Wang et al., 2021 |
| GC | FTO | Alpl, Col1a1, Runx2 | Bone | Promotes osteoblast differentiation by increasing mRNA stability | Feng et al., 2023 |
|  | METTL14 | GPX4 | Bone | Inhibits osteoclast differentiation and promotes bone formation | Deng et al., 2023 |
|  | METTL14 | PTPN6 | Bone | Enhances osteoblast proliferation and differentiation | Cheng et al., 2021 |
|  | METTL14 | GR | Bone | GC inhibits GPX4 to enhance osteoclastogenesis, opposing METTL14 effects | Yang et al., 2021 |
| ER | METTL3 | DMP1 | Cartilage | Enhances chondrocyte hypertrophic differentiation and endochondral ossification | Y. He et al., 2022 |
|  | ALKBH5 | CYP1B1 | Bone, Cartilage | Enhances MSC senescence and impairs cartilage and bone formation | Ye et al., 2023 |
|  | METTL3 | Runx2 | Bone | Promotes osteoblast differentiation by stabilizing Runx2 mRNA | Yan et al., 2020 |
|  | METTL3 | HIF-1α, VEGF-A | Bone | Stimulates osteoblast differentiation and inhibits osteoclast differentiation | Tian et al., 2023 |
|  | METTL14 | Alp, Bglap, Col1α1 | Bone | Stimulates osteoblast proliferation, differentiation, and matrix mineralization | Z. Sun et al., 2021 |
|  | FTO | miR-22-3p | Bone | Promotes osteoblast differentiation by degrading c-Myc mRNA | Zhang et al., 2020 |
|  | METTL3 | CHI3L1 | Bone | Stimulates osteoclast differentiation via NAFTc1 expression | C. Wang et al., 2023b |
|  | METTL14 | miR-103-3p | Bone | Stimulates osteoblast differentiation and m6A methylation of osteogenic markers | Z. Sun et al., 2021 |

**Table 4. Examples of known regulatory connections between components of m6A RNA methylation and calcium dependent pathways.**

| **Pathway** | **m6A RNA Component** | **Pathway Component** | **Target Tissue** | **Cellular Outcome** | **Reference** |
| --- | --- | --- | --- | --- | --- |
| NFAT | METTL3 | Atp6v0d2 | Bone | Impaired osteoclast function by degrading Atp6v0d2 | Li et al., 2020 |
|  | METTL3 | CHI3L1 | Bone | Promotes NFATc1 expression and osteoclast differentiation | C. Wang et al., 2023c |
|  | METTL14 | NFATc1 | Bone | Inhibits osteoclast differentiation by decreasing NFATc1 mRNA stability | J. G. Yang et al., 2023 |
|  | FTO | NFATc1 | Bone | Induces osteoclast differentiation by increasing NFATc1 mRNA stability | Shen et al., 2023 |
|  | FTO | NF-κB | Bone | Facilitates NF-κB binding to NFATc1 promoter, promoting osteoclast differentiation and bone resorption | Zhuang et al., 2021 |
|  | YTHDF2 | NFATc1 | Bone | Degrades NFATc1 mRNA and inhibits osteoclast differentiation | Fang et al., 2021 |
|  | WTAP | miR-29b-3p | Bone | Inhibits NFATc1 function via HDAC4 targeting, promoting osteogenesis | J. Liu et al., 2023 |
| NO | METTL3 | NOS2 | Bone | Decreases NOS2 mRNA stability, inhibiting NO signaling and promoting osteoclast differentiation | Li et al., 2023 |
|  | METTL3 | iNOS | Bone | Increases iNOS mRNA stability, promoting macrophage-induced osteoblast differentiation | Lei et al., 2021 |
| PTH | METTL3 | Pth1r | Bone | Promotes PTH-driven osteoblast proliferation and differentiation | Wu et al., 2018 |
|  | METTL3 | Pth1r | Tooth | Enhances odontoblast proliferation, migration, and differentiation | Sheng et al., 2021 |
